# Comprehensive pharmacogenomic characterization of gastric cancer

**DOI:** 10.1186/s13073-020-0717-8

**Published:** 2020-02-18

**Authors:** Jason K. Sa, Jung Yong Hong, In-Kyoung Lee, Ju-sun Kim, Moon-Hee Sim, Ha Jung Kim, Ji Yeong An, Tae Sung Sohn, Joon Ho Lee, Jae Moon Bae, Sung Kim, Kyoung-Mee Kim, Seung Tae Kim, Se Hoon Park, Joon Oh Park, Ho Yeong Lim, Won Ki Kang, Nam-Gu Her, Yeri Lee, Hee Jin Cho, Yong Jae Shin, Misuk Kim, Harim Koo, Mirinae Kim, Yun Jee Seo, Ja Yeon Kim, Min-Gew Choi, Do-Hyun Nam, Jeeyun Lee

**Affiliations:** 1 Department of Biomedical Sciences, Korea University College of Medicine, Seoul, Republic of Korea; 2 Division of Hematology-Oncology, Department of Medicine, Samsung Medical Center, Sungkyunkwan University School of Medicine, Seoul, Republic of Korea; 3 Department of Surgery, Samsung Medical Center, Sungkyunkwan University School of Medicine, Seoul, Republic of Korea; 4 Department of Pathology, Samsung Medical Center, Sungkyunkwan University School of Medicine, Seoul, Republic of Korea; 5 Institute for Refractory Cancer Research, Samsung Medical Center, Seoul, Republic of Korea; 6 Department of Health Sciences and Technology, Samsung Advanced Institute for Health Science and Technology, Sungkyunkwan University, Seoul, Republic of Korea; 7 Department of Neurosurgery, Samsung Medical Center, Sungkyunkwan University School of Medicine, Seoul, Republic of Korea

**Keywords:** Gastric cancer, Pharmacogenomics, PIK3CA-E542K, RNF11, Gefitinib

## Abstract

**Background:**

Gastric cancer is among the most lethal human malignancies. Previous studies have identified molecular aberrations that constitute dynamic biological networks and genomic complexities of gastric tumors. However, the clinical translation of molecular-guided targeted therapy is hampered by challenges. Notably, solid tumors often harbor multiple genetic alterations, complicating the development of effective treatments.

**Methods:**

To address such challenges, we established a comprehensive dataset of molecularly annotated patient derivatives coupled with pharmacological profiles for 60 targeted agents to explore dynamic pharmacogenomic interactions in gastric cancers.

**Results:**

We identified lineage-specific drug sensitivities based on histopathological and molecular subclassification, including substantial sensitivities toward VEGFR and EGFR inhibition therapies in diffuse- and signet ring-type gastric tumors, respectively. We identified potential therapeutic opportunities for WNT pathway inhibitors in *ALK*-mutant tumors, a significant association between *PIK3CA-*E542K mutation and AZD5363 response, and transcriptome expression of *RNF11* as a potential predictor of response to gefitinib.

**Conclusions:**

Collectively, our results demonstrate the feasibility of drug screening combined with tumor molecular characterization to facilitate personalized therapeutic regimens for gastric tumors.

## Background

Cancer is a complex disease, with profound genomic alterations and diverse cellular hierarchy [1–4]. Advancements in the field of genetics have enabled us to achieve a comprehensive understanding of the tumor molecular structure and the impacts of core oncogenic pathways that are frequently dysregulated [3, 5–7]. However, the development of effective treatments based on molecular characterization of the tumor alone has been increasingly recognized to be limited due to the co-existence of multiple genomic aberrations within a given tumor. To address this challenge, large-scale drug sensitivity profiles of conventional cancer cell-line models have been employed to identify clinically relevant biomarkers that could be therapeutically exploited [8–11]. While these studies have provided unprecedented insights into the biological signaling networks that govern dynamic cellular responses to a broad range of therapeutics, the heterogeneous biological traits of patient-derived tumors hamper the direct application of the current pharmacogenomic atlas in the clinic. We have previously established a compilation of chemical-genetic associations across a wide spectrum of patient-derived tumor cell (PDC) models and demonstrated its clinical feasibility [12, 13]. To further interrogate the dynamics of pharmacogenomic interactions at a single tumor-lineage resolution, we established a library of tumor cell models from surgically resected tumor specimens or ascites-derived tumor cells from gastric cancers and explored potential gene-drug associations for 60 molecularly targeted agents.

Gastric cancer is the third leading cause of cancer-induced mortality worldwide [14–16]. The vast majority of gastric tumors are diagnosed as adenocarcinomas and can be subcategorized into distinct classifications based on molecular, histological, and pathological features [3, 17]. The current standard regimen consists of surgical resection followed by radio-chemotherapy. Although integrated molecular characterization of gastric adenocarcinomas through nationwide efforts of The Cancer Genome Atlas Research Network has led to the identification of major genetic aberrations and oncogenic pathways that contribute to the malignancy of gastric cancer, the clinical application potential of molecular targeted therapy remains obscure. To date, only two molecular target agents, including trastuzumab (anti-HER2 monoclonal antibody) tested in the ToGA trial and ramucirumab (anti-VEGFR monoclonal antibody) investigated in the RAINBOW and REGARD trials have been approved for clinical treatment of metastatic gastric cancer [18–20]. Numerous clinical trials on agents targeting major oncogenic pathways, including HER2 (lapatinib, pertuzumab, trastuzumab-emtansine), EGFR (cetuximab, panitumumab, nimotuzumab, gefitinib), FGFR2 (AZD4547), VEGF (bevacizumab, aflibercept), MET (onartuzumab, rilotuzumab), and PI3K/AKT/mTOR (PAM; ipatasertib, everolimus) have shown disappointing results despite promising preclinical evidences [16]. The current limitations in applicability of molecular-guided therapy are presumably due to inadequate patient stratification and the extensive inter-tumoral heterogeneity of gastric tumors. To address these challenges, we analyzed somatic mutations, copy number alterations, and/or gene expression profiles of 131 gastric tumors as potential predictors of drug sensitivities for 60 anti-cancer compounds to identify molecular determinants that may aid in a paradigm shift towards personalized treatment of gastric cancer.

## Methods

### Gastric tumor specimens and in vitro cell culture

After receiving informed consent, gastric tumor specimens or malignant ascites were obtained from patients undergoing surgery at Samsung Medical Center (SMC) in accordance with the Samsung Medical Center Institutional Review Board. This study was conducted in compliance with all relevant ethical regulations for human specimen research. Portions of the surgical samples were enzymatically dissociated using Liberase™ (Roche) and tumor cells from malignant effusions were collected by centrifugation at 300*g* for 10 min, followed by washing with Dulbecco’s phosphate-buffered saline. Patient-derived tumor cells (PDCs) were cultured in neurobasal medium with N2 and B27 supplements (0.5× each; Thermo Fisher Scientific) and human recombinant basic fibroblast growth factor and epidermal growth factor (20 ng/ml; R&D Systems). Human gastric cancer cell-lines were purchased from the Korean Cell Line Bank. All cell lines were cultured in RPMI 1640 medium supplemented with 10% fetal bovine serum and Antibiotic-Antimycotic (penicillin and streptomycin; Invitrogen) at 37 °C in a humidified atmosphere with 5% CO_2_. PDCs and all cancer cell-lines were tested for mycoplasma contamination.

### Exome sequencing

Tumors were subjected to target exome sequencing using CancerSCAN, a targeted sequencing platform designed at Samsung Medical Center. CancerSCAN covers a range of exonic regions of specific genes that are associated with cancer progression. Genomic DNA was sheared in Covaris S220 sonicator (Covaris) to construct a sequencing library using the SureSelect XT Reagent Kit, HSQ (Agilent Technologies), enriched for target genes. The library was purified and amplified with a barcode tag, and library quality and quantity were determined. Sequencing was carried out using the 100-bp paired-end mode of the TruSeq Rapid PE Cluster kit and TruSeq Rapid SBS kit on a HiSeq 2500 sequencing platform (Illumina). The target exome sequencing data of previous gastric cancer cases were downloaded from the European Genome-phenome Archive (EGAS00001002515).

### Mutation calls

The sequenced reads in FASTQ files were aligned to the human genome assembly (hg19) using the Burrows-Wheeler Aligner. The initial alignment BAM files were subjected to sorting (SAMtools), removal of duplicated read (Picard), local realignment of reads around potential small insertions/deletions, and recalibration of the base quality score (Genome Analysis Toolkit). MuTect was used to generate high-confidence mutation calls. Variant Effector Predictor was used to annotate the called mutations.

### Copy number alteration

ONCOCNV was used to generate estimated copy number alterations in tumor specimens.

### RNA sequencing

RNA-seq libraries were prepared using the Illumina TruSeq RNA Sample Prep kit. Sequenced reads were mapped onto hg19 using the Burrows-Wheeler Aligner. The initial BAM files were sorted and summarized into BED files using SAMtools and bedTools. The BED files were used to calculate the reads per kilobase of transcript per million reads (RPKM) value for each gene, using the DEGseq package.

### Drug screening

PDCs were cultured in serum-free medium, dissociated into single cells, and seeded in 384-well plates at 500 cells/well in duplicate or triplicate for each treatment. The drug panel consisted of 60 molecular target agents targeting oncogenic signals (Additional file 1: Table S1). All drugs were purchased from Selleckchem. PDCs were treated with the drugs in a fourfold or seven-point serial dilution series from 4.88 nM to 20 μM using Janus Automated Workstation (PerkinElmer). After 7 days of incubation, cell viability was analyzed using ATP monitoring system based on firefly luciferase (ATPLite™ 1step; PerkinElmer). Viable cells were estimated using EnVision Multilabel Reader (PerkinElmer). Control cells treated with dimethyl sulfoxide (DMSO) vehicle were used to calculate relative cell viability for each plate and to normalize the data on a per-plate basis. Dose response curve fitting was performed using GraphPad Prism 5 (GraphPad) and was evaluated by measuring the area under curve (AUC). In brief, each plate was normalized to the mean value from the seven serial conditions compared with DMSO control. The AUC of each curve was determined using GraphPad Prism (GraphPad Software), ignoring regions defined by fewer than two peaks. Non-convergence or ambiguous curves were excluded in every analysis. Two identical PDCs and cancer cell-lines were screened every month to validate and confirm the preservation of chemical activities of our drug library and high-throughput drug screening platform. Screening plates were subjected to quality control measurement using *z*-factor scores, comparing both negative and positive control wells [21].

### Pharmacogenomic interactions of genetic variations

A list of genetic variations, including single nucleotide variations, small insertions, small deletions, and copy number alterations, were considered to evaluate drug response interactions. For each drug candidate, drug sensitivity data (AUC) were analyzed by comparing tumors with the selected genomic alterations to those without using the Wilcoxon rank-sum test. Samples with unknown status of a given alteration were excluded from the analysis.

### Elastic net regression model-based analysis

We selected 41 gastric cancer cases with available RNA-seq and drug response data. The input variables for the elastic net regression model-based analysis consisted of gene expression profiles with genomic alterations including mutations and copy number alterations for tumors whose genome data were available. We then trained the standard elastic net regression using the *glmnet* R package by combining input features and comparing to individual drugs response. Afterwards, we employed bootstrapping strategy for 100 times to extract reliable and robust candidate features. During each bootstrapping step, we randomly selected 80% of the tumors for feature extraction. For each feature, the time of its appearance out of 100 bootstrapping and its average weight were used as the final assessment.

### Cell establishment and growth assessment against gefitinib

Gastric cancer cell-lines were transiently transfected with 10 nM of siRNF11 using 6 μL of HiPerFect transfection reagent (Qiagen). Next day, the cells were seeded in 96-well plates at 5000 cells/well, allowed to adhere overnight, and treated with gefitinib for 72 h. Cell proliferation inhibition was determined using CellTiter-Glo Luminescent Cell Viability Assay (Promega) according to the manufacturer’s protocol. The detected luminescent signals were used to calculate the percentage of surviving cells and to obtain AUC values.

### Cellular growth assessment against AZD5363

Gastric cancer cell-lines with *PIK3CA* mutation (E542K or E545K) or wild-type have been seeded in 96-well plates at 5000 cells per well, allowed to adhere overnight and treated with various concentrations (0.3 or 1 μM) of AZD5363 for 72 h, and the cell viability was determined using the Cell Titer Glo.

### siRNA sequences

siRNA constructs for *RNF11* (siRNF11#1: 5′-ACATCTCCCTGCTTCACGAC-3′ and siRNF11#2: 5′-GGAAGAGAUGGAUCAGAAA-3′) and control (siControl: 5′-TAGCGACTAAACACATCAA-3′) were used in this study.

### Immunoblot analysis

Total cell extracts were prepared using lysis buffer (20 mM HEPES [pH 7.4], 1% Triton X-100, 1 mM EDTA, 1 mM MgCl_2_, 150 mM NaCl, 10% glycerol, protease inhibitor, and phosphatase inhibitor cocktail [Invitrogen]). Protein concentrations were determined using micro-BCA protein reagent (Pierce Biotechnology). Thirty micrograms of total proteins were separated by sodium dodecyl sulfate polyacrylamide gel electrophoresis and transferred onto nitrocellulose membranes with 0.2-μm pore size (Whatman). The membranes were incubated with antibodies against phospho-AKT (Ser473) (#4060, 1:1000; Cell Signaling Technology (CST); RRID: AB_2315049), AKT (#9272, 1:1000; CST; RRID: AB_329827), phospho-mTOR (Ser2448) (#2971, 1:1000; CST; RRID: AB_330970), mTOR (#2972, 1:1000; CST; RRID: AB_330978), phospho-S6 ribosomal protein (Ser2235/236) (#2211, 1:1000; CST; RRID: AB_331679), S6 ribosomal protein (#2217, 1:1000; CST; RRID: AB_331355), phospho-4E-BP1 (Thr70) (#13396, 1:1000; CST; RRID: AB_2798206), 4E-BP1 (#9644, 1:1000; CST; RRID: AB_2097841), phospho-EGFR (Tyr1068) (#3777, 1:1000; CST; RRID: AB_2096270), EGFR (#2646, 1:1000; CST; RRID: AB_2230881), RNF11 (ab154831, 1:1000; Abcam), or β-actin (AC-15, 1:5000; Sigma; RRID: AB_476692). The ECL method (Invitrogen) was used for protein detection.

### Statistical analysis

Data are presented as the mean ± standard deviation (SD). All statistical analyses were conducted by either Wilcoxon rank-sum test (two-sided), Pearson’s correlation coefficient test, or Fisher’s exact test (two-sided) as relevant. All statistical analyses were conducted using the R software (https://www.r-project.org) or GraphPad Prism.

## Results

### Mutational landscape of gastric cancer

**Fig. 1:**
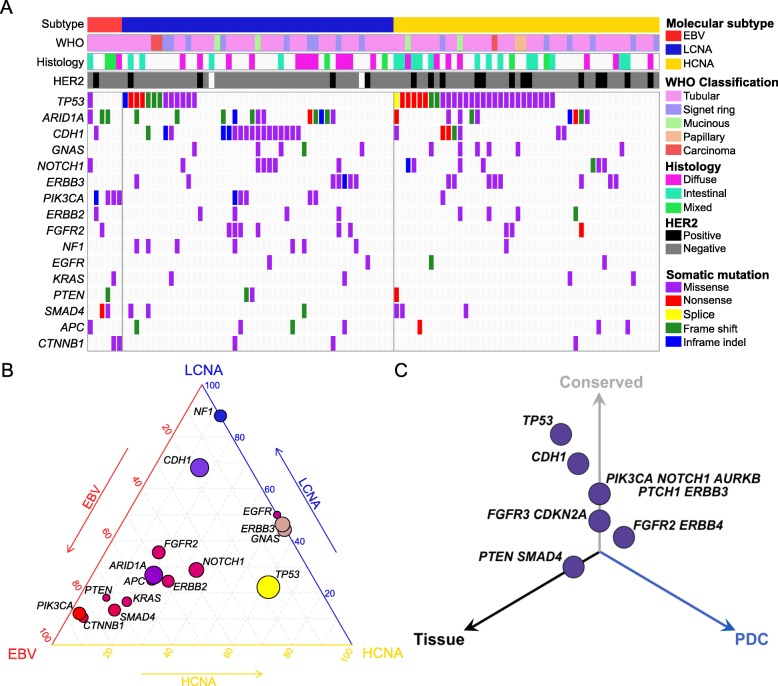
Mutational landscape of gastric cancer. **a** Mutational landscape of gastric cancers based on molecular subclassification; EBV-positive, LCNA, and HCNA tumors. All mutations with variant allele frequency of > 5% and depth of > 20 reads are shown. **b** Ternary diagram depicting mutation frequencies in EBV-positive, LCNA, and HCNA tumors. The size of each node represents the number of tumors with the respective mutation, and the color spectrum indicates its relative frequency. **c** Three-dimensional bubble plot showing the frequency of non-synonymous cancer-driver genomic mutations exclusively in tissue (black, left axis), in PDCs (blue, right axis), or in both (gray, upper axis). The position of each dot or mutation is located on the quadrant based on its shared or private rate between primary tumor tissues and matched PDCs, and the distance reflects the number of cases that harbor respective mutation

To explore the dynamics of pharmacogenomic interactions in gastric cancers, we generated 131 surgically resected gastric tumor specimens or malignant ascites (Additional file 2: Table S2). To determine genomic variations, including single-nucleotide variants (SNVs), small insertions/deletions (Indels), and copy number alterations (CNAs; segments of the genome that are either amplified or deleted), 102 tumor specimens were subjected to targeted massively parallel sequencing, covering the full coding exons of major cancer-driver genes (Additional file 3: Table S3). Mutations with variant allele frequency of > 5% and > 20 reads were considered (Additional file 4: Table S4). Forty-one tumors were subjected to whole-transcriptome sequencing to curate gene expression profiles. Tumors were classified into four subgroups based on molecular profiles: Epstein-Barr Virus (EBV)-positive, microsatellite instability (MSI)-high, high copy number alterations (HCNA), and low copy number alterations (LCNA) (Additional file 5: Figure S1). HCNA tumors exhibited enrichment of *TP53* mutation, whereas LCNA and EBV-positive tumors were marked by high prevalence of *CDH1* and *PIK3CA* mutations, respectively (Fig. 1a, b) [3, 22]. Notably, LCNA tumors showed recurrent genetic aberrations of *NF1*, suggesting potential therapeutic opportunities for RAS/MAPK-targeted therapies [23]. Furthermore, the HCNA group demonstrated a significantly higher rate of HER2-positive tumors than the other types. Compared with other large-scale gastric cancer datasets, our cohort constituted comparable levels of major gastric cancer-driver genes, including somatic mutations of *TP53*, *ARID1A*, *PIK3CA*, and *APC* (Additional file 5: Figure S2) [3, 24, 25]. On note, our cohort harbored higher frequency of *CDH1*-mutant tumors and we suspect that this could be due to the higher number of LCNA or genomically stable tumors, which are marked by enrichment of *CDH1* mutation (Additional file 5: Figure S2).

Compared with traditional long term-cultured cancer cell-line models, PDCs recapitulate the molecular properties and biology of diseases more precisely, prompting their feasibility as a reliable model system for evaluating the potential clinical response to various therapeutics [26–28]. One hundred twenty-eight gastric PDCs were cultured under serum-free conditions and were used in a systematic drug sensitivity screening of 60 compounds that target major oncogenic pathways, including receptor tyrosine kinases (RTKs), poly(ADP-ribose) polymerase (PARP), and histone deacetylase (HDAC) (Additional file 1: Table S1 and Additional file 5: Figure S3A). Drug sensitivities were determined based on the area under curve (AUC) of the dose-response curve after 7 days of treatment (Additional file 6: Table S5). For most compounds, the PDCs exhibited a wide range of sensitivities. Interestingly, t-stochastic neighbor embedding (tSNE) analysis revealed a hierarchical clustering of the RTK inhibitors, confirming the validity of our systematic screening procedure and the target inhibitor quality assessment (Additional file 5: Figure S3B). A number of PDC lines were further subjected to exome sequencing to interrogate whether they retained the spectrum of genomic aberrations observed in the matched tumor specimens. Consistent with previous findings, major cancer-driver alterations, including *TP53*, *CDH1*, *PIK3CA*, *ERBB3*, and *FGFR3* mutations were highly preserved in the PDCs (Fig. 1c) [12, 13]. Collectively, these results suggest that PDCs recapitulate tumor molecular properties and can serve as proxies for comprehensive pharmacogenomic analyses in gastric tumors.

### Subgroup-specific drug sensitivities based on molecular, histological, and pathological classifications

**Fig. 2:**
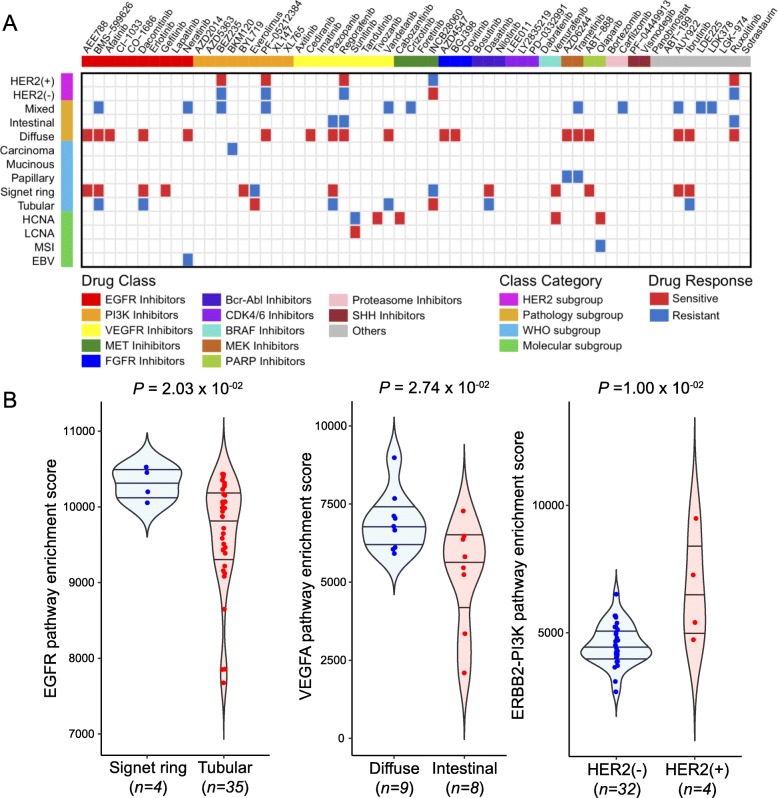
Gastric cancer subgroup-specific drug sensitivity. **a** Heatmap representation of drug sensitivities in gastric cancer based on molecular, histological, and pathological subclassification. Only significant associations are marked based on sensitivity (red) or resistance (blue). Drugs were clustered based on their known target classes. **b** Violin plots demonstrating pathway enrichment scores of each corresponding pathway. The activity scores were measured using ssGSEA. Horizontal lines within the violin plots represent 0.25, 0.50, and 0.75 quantiles. *P* values in **a**, **b** were derived from two-sided Wilcoxon rank-sum tests

As gastric cancers can be subcategorized based on molecular, histological, and pathological features, we evaluated the pharmacological landscape of distinct subgroups of gastric PDCs within each subtype category. Overall, we observed a wide range of drug sensitivities within each class, demonstrating the highly heterogeneous nature of gastric PDCs (Additional file 5: Figure S4A and Additional file 7: Table S6). Molecular variations across each subcategory potentially contribute to dynamic drug responses. Of note, diffuse-subtype tumors were highly sensitive to multiple RTK inhibitors targeting VEGFR, PDGFR, and FGFR pathways (cediranib, vandetanib, pazopanib, regorafenib, AZD4547, and BGJ398), whereas mixed types were mostly resistant (Fig. 2a). Furthermore, PDCs from signet-ring type demonstrated considerable sensitivities to EGFR inhibitors, including AEE788, afatinib, dacomitinib, and gefitinib, which were comparatively less potent in tubular subtype. HER2-positive tumors were highly sensitive to PAM compounds, including BEZ235 and PF-05212384, when compared with HER2-negative tumors. Consistent with the heterogeneous pharmacological behaviors of gastric PDCs, pathway enrichment analysis revealed enrichments of the EGFR, VEGFR, and ERBB2-PI3K pathways in signet-ring, diffuse, and HER2-positive tumors, respectively (Fig. 2b). Moreover, tumors with high chromosomal instability were considerably more sensitive to olaparib. PARP inhibition therapy has demonstrated significant therapeutic success in patients diagnosed with either advanced ovarian or metastatic breast cancer with germline BRCA1/2 mutations [29, 30]. Therefore, we sought to evaluate the prevalence of *BRCA1/2* mutations in each molecular gastric tumor subtype. Notably, *BRCA2* mutation constituted a significant proportion in HCNA tumors, suggesting clinical application potential of olaparib for patients with high chromosomal instability and *BRCA2* variation (Additional file 5: Figure S4B). Furthermore, we found that majority of the *BRCA2*-mutant tumors were *CDH1* wild-type (Additional file 5: Figure S4B). Together, these results underscore the significance of systematic drug sensitivity screening to guide subtype-specific targeted therapeutic opportunities in gastric cancers.

### Pharmacogenomic landscape of gastric cancer

**Fig. 3:**
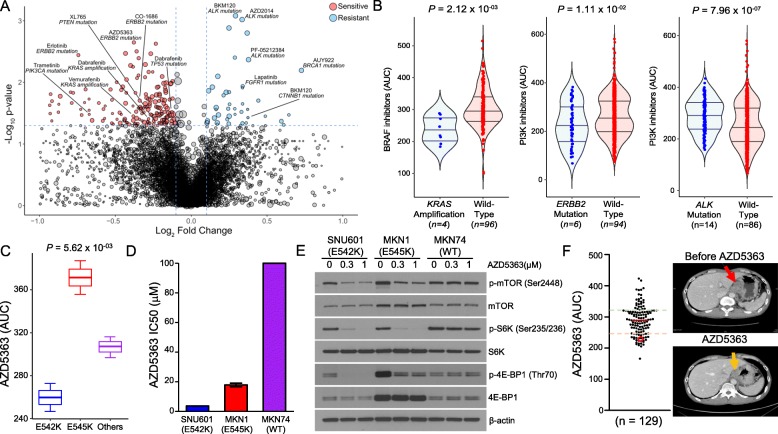
Pharmacogenomic interactions in gastric cancer. **a** Volcano plot representation of pharmacogenomic interactions in gastric cancer with fold-change drug comparison (*x*-axis) and its significance (*y*-axis). Each node represents a single genomic alteration-drug interaction, and the size is proportional to the number of tumors with the respective genomic variation. **b** Violin plots of drug AUC values for tumors with thegenomic alteration compared to those without from selected gene-drug interactions. Horizontal lines within the violin plots represent 0.25, 0.50, and 0.75 quantiles. **c** Box plots of AZD5363 AUC values among tumors with different *PIK3CA* variations. Box plots span from the first to third quartiles, and the whiskers represent the 1.5 interquartile range. **d** Cell proliferation assay of gastric cancer cell-lines. **e** Effects of AZD5363 on the PI3K/AKT/mTOR signaling pathway in gastric cancer cell-lines with different mutations of *PIK3CA* or the wild-type gene. **f** Scatter plot of AZD5363 AUCs in our cohort (left panel). The AUC of the PDC that was isolated from the indicated patient (right panel) is highlighted in a red circle. Dotted green and orange horizontal lines represent relative resistance and sensitivity, respectively. T_1_-weighted contrast-enhanced magnetic resonance images of tumor samples from the gastric cancer patient who received AZD5363 treatment. The red arrow indicates measurable or progressed tumor; the orange arrow represents partial response. *P* values in **a**, **b** were derived from two-sided Wilcoxon rank-sum tests, the *P* value in **c** from one-way ANOVA

Genomic variations, including somatic mutations and copy number alterations, can be employed as reliable biomarkers for predicting clinical response to targeted therapy [31–34]. To identify genomic correlates of pharmacological sensitivity in gastric tumors, we evaluated individual drug sensitivity profiles of PDC lines against each genomic or molecular aberration (Fig. 3a and Additional file 8: Table S7). Notably, tumors with *KRAS* amplification were considerably more sensitive to BRAF inhibitors, including vemurafenib and dabrafenib, whereas *ERBB2*-mutant tumors were therapeutically more susceptible to both EGFR (CO-1686 and erlotinib) and PI3K inhibitors (AZD5363, AZD2014, and everolimus). Somatic mutation in *FGFR2* conferred increased sensitivity to multi-targeted tyrosine kinase inhibitors, including FGFR- and VEGFR-targeting compounds (Additional file 5: Figure S5). Previous studies have presented molecular rationales for treating *ALK*-mutated tumors with PAM pathway inhibitors [35–37]. However, we discovered that mutation in the ALK receptor tyrosine kinase (*ALK*) gene was significantly associated with global resistance to a broad range of therapeutics, including those targeting the PI3K-AKT-mTOR (PAM) pathway (e.g., BKM120, AZD2014, PF-05212384) (Fig. 3b). To identify alternative therapeutic avenues for treating *ALK*-mutated tumors, we conducted a genome-wide comparative transcriptome analysis of *ALK*-mutant and *ALK*-wild-type tumors. Interestingly, *LEF1* was strongly activated in tumors with *ALK* mutation, and single sample gene set enrichment analysis (ssGSEA) consistently demonstrated upregulation of the WNT signaling pathway (Additional file 5: Figure S6). Collectively, these results suggest potential therapeutic benefits of WNT-mediated therapy in patients with *ALK*-mutated tumors.

Somatic mutations in *PIK3CA*, which encodes the catalytic subunit of the phosphatidylinositol 3-kinase (PI3K) complex, have been detected in a broad spectrum of tumor types, including gastric cancer [3, 38–40]. These mutations promote the activation of proto-oncogenic signaling pathways, rendering cells susceptible to malignant transformation. Furthermore, previous studies have shown that *PIK3CA* mutation is a key molecular determinant to AKT inhibition response in gastric cancer cell-lines [41]. Although there are multiple variations of *PIK3CA* mutation, the “hot-spot” mutations are located within the helical (exon 9) or kinase (exon 20) domains [42]. While previous studies have investigated the potential associations between various *PIK3CA* mutations and clinical prognosis [43–45], prediction of pharmacological vulnerability based on these mutations remains elusive. Interestingly, when we evaluated drug sensitivities to PI3K compounds in *PIK3CA*-mutant tumors, we found that SNVs that lead to E542K amino acid substitution induced the most robust susceptibility to an AKT inhibitor (AZD5363) compared to other *PIK3CA* mutations (Fig. 3c). To functionally validate and explore the AZD5363 and *PIK3CA-*E542K association and its biological effects, we assessed therapeutic efficacy of AZD5363 in *PIK3CA*-E542K-mutant, *PIK3CA-*E545K-mutant, and *PIK3CA* wild-type gastric cell-lines. Consistent with the previous findings in PDC models, cytotoxic activity of AZD5363 was the most significant in *PIK3CA-*E542K-mutant tumor cells (Fig. 3d and Additional file 5: Figure S7A). Immunoblot analyses of PI3K pathway downstream effectors, including phosphorylation of mTOR, S6K, and 4E-BP1, demonstrated robust inhibition in the presence of AZD5363 treatment (Fig. 3e). Moreover, combinational treatment of AZD5363 with Taxol further increased apoptotic activities (Additional file 5: Figure S7B). Our findings were further corroborated by clinical data; PDC progeny from a gastric cancer patient with *PIK3CA*-E542K mutation exhibited profound response to AZD5363, marked by partial clinical response (Fig. 3f). Collectively, these findings suggest the clinical feasibility of patient-derived pharmacogenomics as a potential predictor in patient enrichment trials.

### Identification of molecular determinants that dictate drug sensitivity to gefitinib

**Fig. 4:**
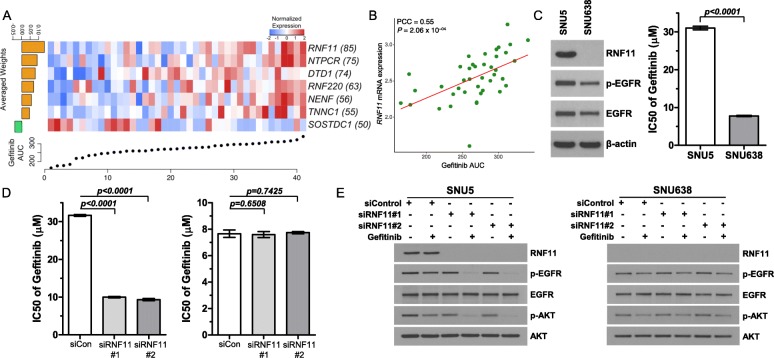
Transcriptome correlates of gefitinib sensitivity. **a** Elastic-net regression results of transcriptome features that predict pharmacological response to gefitinib. The bottom scatter plot represents drug response for gefitinib-treated tumors. The upper heatmap shows the top extracted features in the model. The left bar graph shows the averaged weight of each predictive feature. The number of appearances in 100 bootstraps is indicated in parentheses. **b** Scatter plot revealing a linear correlation between gefitinib AUC and *RNF11* transcriptome expression. Correlation coefficients and *P* values were obtained by Pearson correlation analysis. **c** Immunoblot analysis of RNF11, p-EGFR, EGFR in gastric cancer cell-lines. β-Actin was used as a loading control (left panel). Cell proliferation assay in EGFR-activated gastric cancer cell-lines (right panel). Cancer cells were exposed to gefitinib for 72 h, and then, cell viability was measured. **d** Gastric cancer cell-lines with high (SNU5; left panel) and low (SNU638; right panel) RNF11 expression were transiently transfected with 10 nM of siRNF11 and treated with gefitinib for 72 h the next day. The results are represented as the mean ± SD of triplicate wells and are representative of three independent experiments. **e** Immunoblot analysis of EGFR signaling-related molecules, including p-EGFR, EGFR, p-AKT, and AKT in gastric cancer cell-lines that were transiently transfected with 10 nM of siRNF11 and treated with gefitinib for 4 h the next day. *P* values in **c**, **d** were derived from two-sided Student’s *t* tests

A substantial number of studies have demonstrated that *EGFR* overexpression has been correlated with more malignant phenotypic state and dismal clinical outcomes in gastric cancer patients, suggesting *EGFR* as a therapeutically exploitable target [46–51]. We have previously demonstrated the clinical feasibility of drug screening-guided precision therapy and initiated a prospective evaluation in gastric cancer patients to examine potential responses to sunitinib, imatinib, and gefitinib [12] (National Clinical Trial [NCT] #03170180). Therefore, we sought to identify potential molecular or genomic determinants that could aid in design of effective EGFR targeted therapy, specifically utilizing gefitinib, in treating gastric cancer patients. Previous studies have shown that gene expression profiles can be applied to elucidate the biological mechanisms that underlie complex cellular signaling pathways that are associated with drug response [12, 13, 52–55]. Therefore, we employed elastic-net regression model-based analysis using gene expression profiles combined with previously known drug targets, protein-protein interaction networks, and genomic features. As a result, we identified multiple transcriptome molecules that were highly associated with gefitinib response, including *RNF11*, *NTPCR*, and *RNF220* (Fig. 4a). Among these, the transcriptional expression level of *RNF11* demonstrated the most robust correlation (Fig. 4b). Moreover, *RNF11* showed direct correlations with other EGFR inhibitors, including AEE788, dacomitinib, and lapatinib as well (Additional file 5: Figure S8). RNF11 encodes ring finger protein 11, and earlier studies have postulated that *RNF11* interacts with SARA and ESCRT-0 subunits STAM2 and Eps15b to delay the degradation of EGF-activated EGFR [56]. Interestingly, small interfering RNA (siRNA)-mediated knockdown of *RNF11* in two different RNF11^high^ gastric cancer cells (SNU5 and NCI-N87) conferred increased sensitivities to gefitinib (Fig. 4c, d and Additional file 5: Figure S9A). Consistently, silencing of *RNF11* combined with gefitinib attenuated phosphorylation level of EGFR and its downstream molecule, phospho-AKT, further corroborating that *RNF11* has potential as a molecular predictor of intrinsic resistance to EGFR inhibitors (Fig. 4e and Additional file 5: Figure S9B). Overall, our results provide a therapeutically exploitable genomic marker of drug sensitivity that may aid in the design of future biomarker-driven clinical trials in EGFR-targeted therapy.

## Discussion

The fundamental principle of precision oncology is that molecular characterization of the tumor enables optimal patient-tailored therapy [57, 58]. With the exponential increase in systematic tumor genome sequencing efforts in recent years, molecular aberrations that govern essential cellular programs that are therapeutically exploitable have been identified [34, 59, 60]. However, substantial evidence highlights the current limitations in predicting successful clinical therapies on the sole basis of computational data [61, 62]. Therefore, systematic evaluation of tumor genome and simultaneous assessment of drug sensitivities have become the next step towards addressing precision oncology therapy. In the present study, we generated a drug sensitivity dataset based on 131 patient-derived tumor specimens that was molecularly annotated to explore dynamic pharmacogenomic interactions in gastric cancers. Consistent with previous genomic characterization of gastric cancers based on molecular classification, we observed enrichments of *PIK3CA* and *TP53* somatic mutations in EBV-positive and HCNA-subtype gastric tumors, respectively, whereas *CDH1* and *NF1* aberrations were evident in genomically stable (LCNA) tumors. We also identified lineage-specific drug associations, for example, between EGFR inhibitors and signet-ring tumors, and VEGFR compounds and diffuse-type tumors. Consistent herewith, the recent REGARD clinical trial revealed that the VEGFR2 antagonist ramucirumab showed prominent clinical benefits in diffuse tumors compared with intestinal-type tumors based on subgroup analysis [20]. Furthermore, we found that tumors with high chromosomal instability (HCNA) were considerably more sensitive to PARP inhibition treatment and harbored higher frequency of *BRCA2* mutations. Targeting BRCA1/2-deficient cancers using PARP inhibitors has been the archetype of synthetic lethality based on the inhibition of DNA damage repair (DDR) pathway. The first-in-class PARP inhibitor, olaparib, has been the most extensively studied compound of DDR inhibitors, and the therapeutic landscape of olaparib has been rapidly expanding [63]. Previous phase II clinical trial showed that olaparib with paclitaxel demonstrated greater overall survival benefits in patients with metastatic gastric cancer, specifically those with low ataxia telangiectasia mutated (ATM) expression level [64]. Unfortunately, the subsequent phase III trial failed to attain its primary objective [65]. The current limitation on PARP inhibition therapy necessitated assessment of additional biomarkers to achieve successful clinical outcomes in gastric cancer. In such context, our results suggest that patients with high chromosomal instability and *BRCA2* mutation could potentially benefit from olaparib treatment.

Moreover, through large-scale pharmacogenomic analyses, we suggested inhibition of WNT signaling as a therapeutic option for *ALK*-mutant tumors. Furthermore, we found significant therapeutic vulnerabilities of *PIK3CA-*E542K mutant tumors to AKT inhibition therapy. Of note, we have previously performed the first and largest prospective molecular-guided targeted therapy in patients with gastric cancer, aligned with eight pre-specified genomic biomarkers and ten independent biomarker-associated clinical trials (The VIKTORY Umbrella Trial) [66]. Consistent with previous observations, we discovered that patients with *PIK3CA*-E542K mutant tumors demonstrated the most robust response to AKT inhibitors when compared with patients harboring E545K, E545G, E453K, or other mutant type tumors. Our findings were further experimentally validated where AZD5363 demonstrated potent cytotoxic activities in *PIK3CA-*E542K-mutant gastric cancer cell-lines, subsequently downregulating PI3K pathway encoding molecules, including phosphorylation of mTOR, S6K, and 4E-BP1. These results collectively suggest that the current pharmacogenomic atlas of PDC models support the clinical feasibility of molecular-guided targeted therapy in hopes of expediting personalized treatment.

Lastly, we identified molecular determinants, including *RNF11*, of the therapeutic response to gefitinib treatment. *RNF11* has been presented as a proto-oncogene, delaying the degradation of EGF-activated EGFR signaling pathway components. Previous phase II and III clinical trials using EGFR-targeted agents (cetuximab, panitumumab, nimotuzumab, gefitinib) in gastric cancer were terminated due to insignificant survival advantage [49, 67–69]. However, recent reports suggested the clinical feasibility of EGFR-mediated therapy, especially for patients with *EGFR* amplification in gastric tumors, and revealed potential mechanisms underlying EGFR therapeutic resistance [70]. Notably, we discovered that signet-ring cell-type tumors demonstrated considerable sensitivities to EGFR inhibitors, while tubular types were widely resistant. Conversely, siRNA-mediated knockdown of *RNF11* sensitized tumor cells, including tubular-type tumors to gefitinib. These results suggest a potential combinational strategy to circumvent EGFR-mediated therapeutic resistance.

In conclusion, integration of tumor genome and drug sensitivity data is the next step towards precision oncology therapy. As molecular target agents, including trastuzumab and ramucirumab, are currently being used in combination with cytotoxic chemotherapeutic compounds (5FU/CDDP and paclitaxel, respectively) to treat gastric cancer patients [18, 19], we believe that the results in this study provide opportunities to design effective clinical trials and combinational therapeutic strategies in hopes of facilitating clinical application towards personalized treatment in gastric cancer.

## Conclusions

In summary, we have established a systematic framework for genetic prediction of anticancer drug response using patient-derived resources that are molecularly, clinically, and pharmacologically annotated. Through comprehensive pharmacogenomic analyses, we identified lineage-specific drug sensitivities and gene-drug interactions that are well represented within clinical context, including VEGFR inhibitors with diffuse-type tumors and *PIK3CA-*E542K mutation with AKT inhibition therapy. Furthermore, we suggested *RNF11* expression as a predictive biomarker for gefitinib treatment in gastric cancer.

## Supplementary information

**Additional file 1: Table S1.** List of 60 drugs that were used in the drug sensitivity analysis. Drugs have been annotated based on their chemical and/or generic name, target molecule and clinical phase.

**Additional file 2: Table S2.** Clinical annotations of 131 gastric cancers used in the drug screening and/or genomic characterization.

**Additional file 3: Table S3.** List of major cancer-driver genes and sequencing library information for CancerSCAN.

**Additional file 4: Table S4.** Mutational landscape of gastric cancer.

**Additional file 5: Figure S1.** Unsupervised clustering of gastric cancers. **Figure S2.** Frequency of major gastric cancer-driver gene alterations. **Figure S3.** Pharmacological landscape of gastric cancers for 60 molecular-targeted compounds. **Figure S4.** Subgroup-specific drug sensitivity among gastric cancers. **Figure S5.** Gene-drug associations among gastric cancers. **Figure S6.** Pathway enrichment analysis between *ALK-*mutant and *ALK* wild-type tumors. **Figure S7.** Pharmacological effects of *PIK3CA* mutations on AZD5363 and Taxol combination treatment. **Figure S8.** Correlations between *RNF11* mRNA expression level and EGFR inhibitors. **Figure S9.** siRNA-mediated knockdown of *RNF11* promotes therapeutic sensitivity to gefitinib.

**Additional file 6: Table S5.** Area Under the Curve (AUC) values for 60 drugs in 129 PDCs.

**Additional file 7: Table S6.** Tumor type-specific drug associations. Wilcoxon rank-sum test was applied to determine the relative difference of drug sensitivity between individual tumor type and the rest.

**Additional file 8: Table S7.** Pharmacogenomic interactions using integrative analysis of drug sensitivity results (AUC) and genomic alterations. The statistical significance was calculated using Wilcoxon rank-sum test.

## Data Availability

All newly sequenced data have been deposited in the European Genome-phenome Archive (EGA) under accession EGAS00001004106 [71].
