# Supplementary material for: Comprehensive pharmacogenomic characterization of gastric cancer
Source: Genome Med. 2020 Feb 18;12:17. doi: 10.1186/s13073-020-0717-8 (PMC7029441; doi:10.1186/s13073-020-0717-8)
Supplement: Supplementary file 5 — Additional file 5: Figure S1. Unsupervised clustering of gastric cancers. Figure S2. Frequency of major gastric cancer-driver gene alterations. Figure S3. Pharmacological landscape of gastric cancers for 60 molecular-targeted compounds. Figure S4. Subgroup-specific drug sensitivity among gastric cancers. Figure S5. Gene-drug associations among gastric cancers. Figure S6. Pathway enrichment analysis between ALK-mutant and ALK wild-type tumors. Figure S7. Pharmacological effects of PIK3CA mutations on AZD5363 and Taxol combination treatment. Figure S8. Correlations between RNF11 mRNA expression level and EGFR inhibitors. Figure S9. siRNA-mediated knockdown of RNF11 promotes therapeutic sensitivity to gefitinib. [file 13073_2020_717_MOESM5_ESM.pptx]

## Slide 1
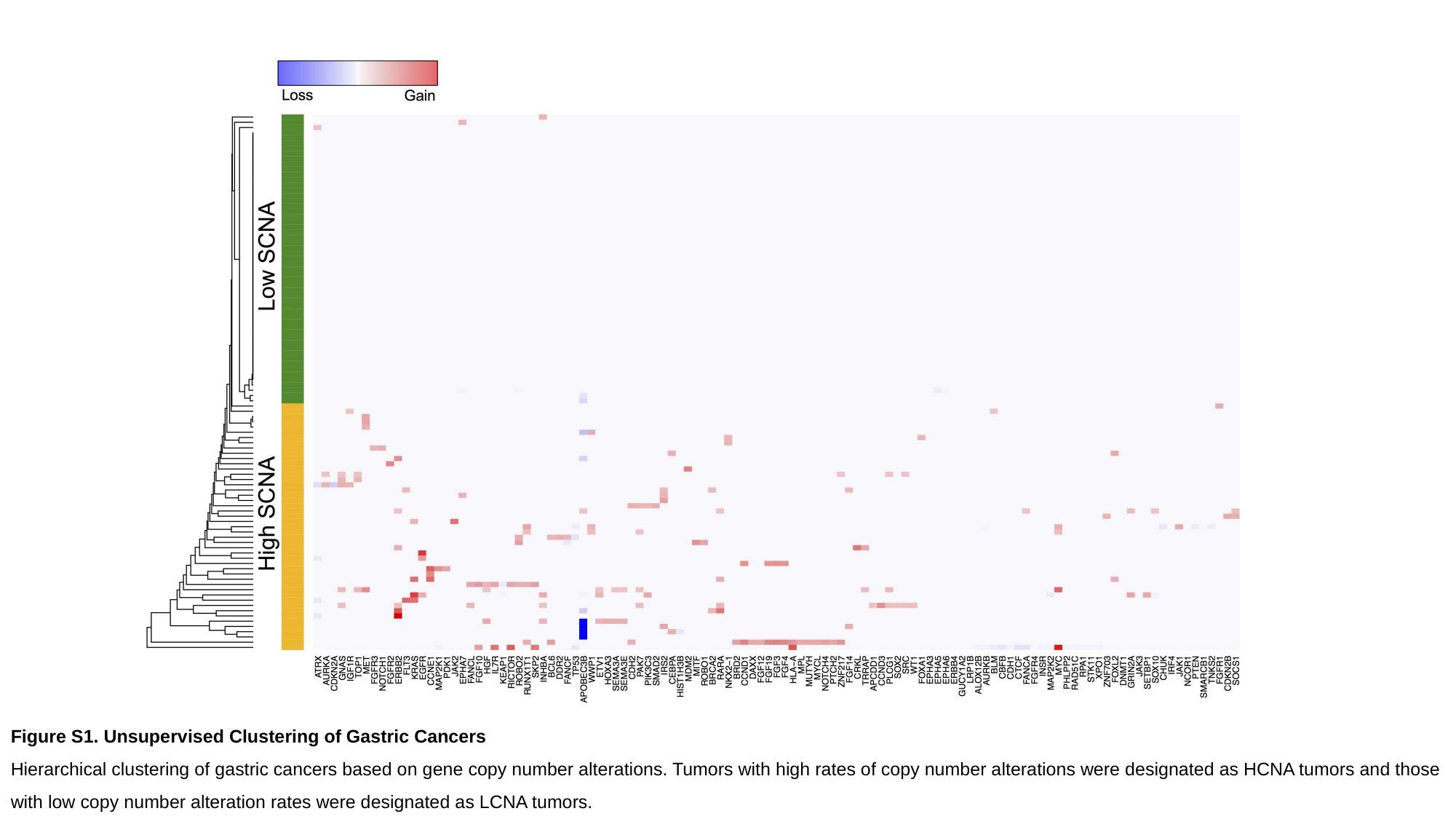

Figure S1. Unsupervised Clustering of Gastric Cancers
Hierarchical clustering of gastric cancers based on gene copy number alterations. Tumors with high rates of copy number alterations were designated as HCNA tumors and those with low copy number alteration rates were designated as LCNA tumors.

## Slide 2
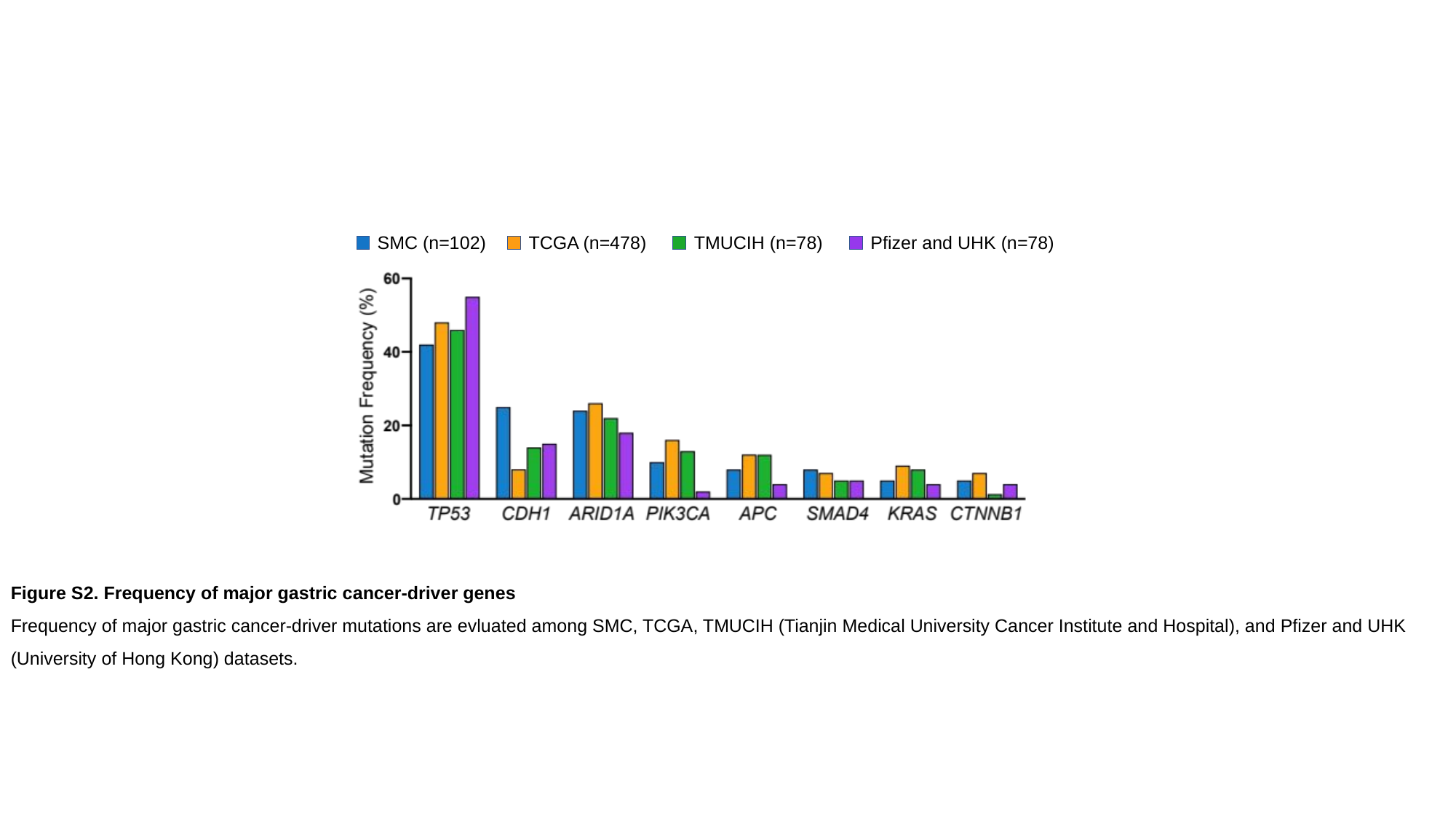

SMC (n=102)
TCGA (n=478)
TMUCIH (n=78)
Pfizer and UHK (n=78)
Figure S2. Frequency of major gastric cancer-driver genes
Frequency of major gastric cancer-driver mutations are evluated among SMC, TCGA, TMUCIH (Tianjin Medical University Cancer Institute and Hospital), and Pfizer and UHK (University of Hong Kong) datasets.

## Slide 3
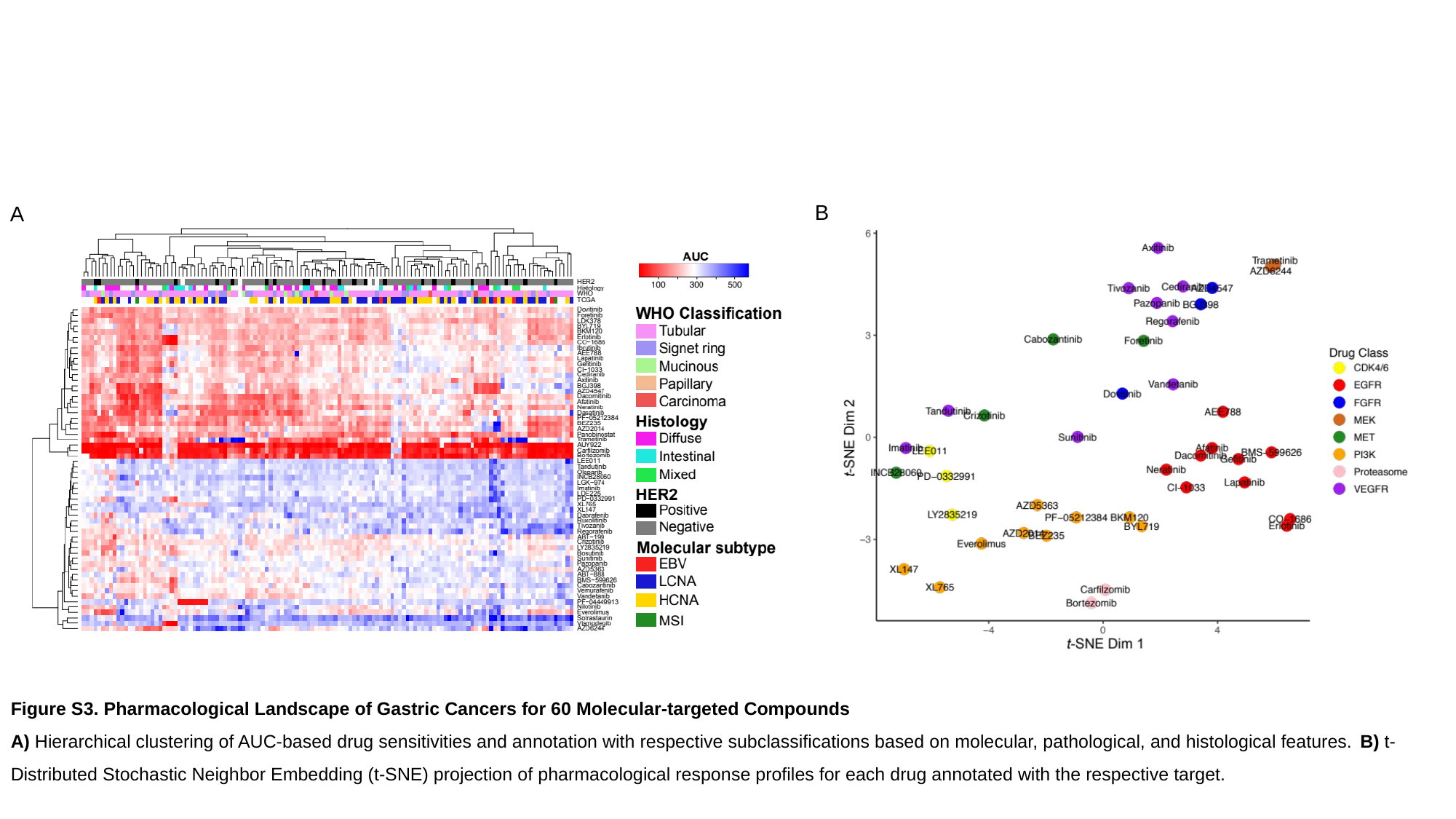

B
A
Figure S3. Pharmacological Landscape of Gastric Cancers for 60 Molecular-targeted Compounds
A) Hierarchical clustering of AUC-based drug sensitivities and annotation with respective subclassifications based on molecular, pathological, and histological features. B) t-Distributed Stochastic Neighbor Embedding (t-SNE) projection of pharmacological response profiles for each drug annotated with the respective target.

## Slide 4
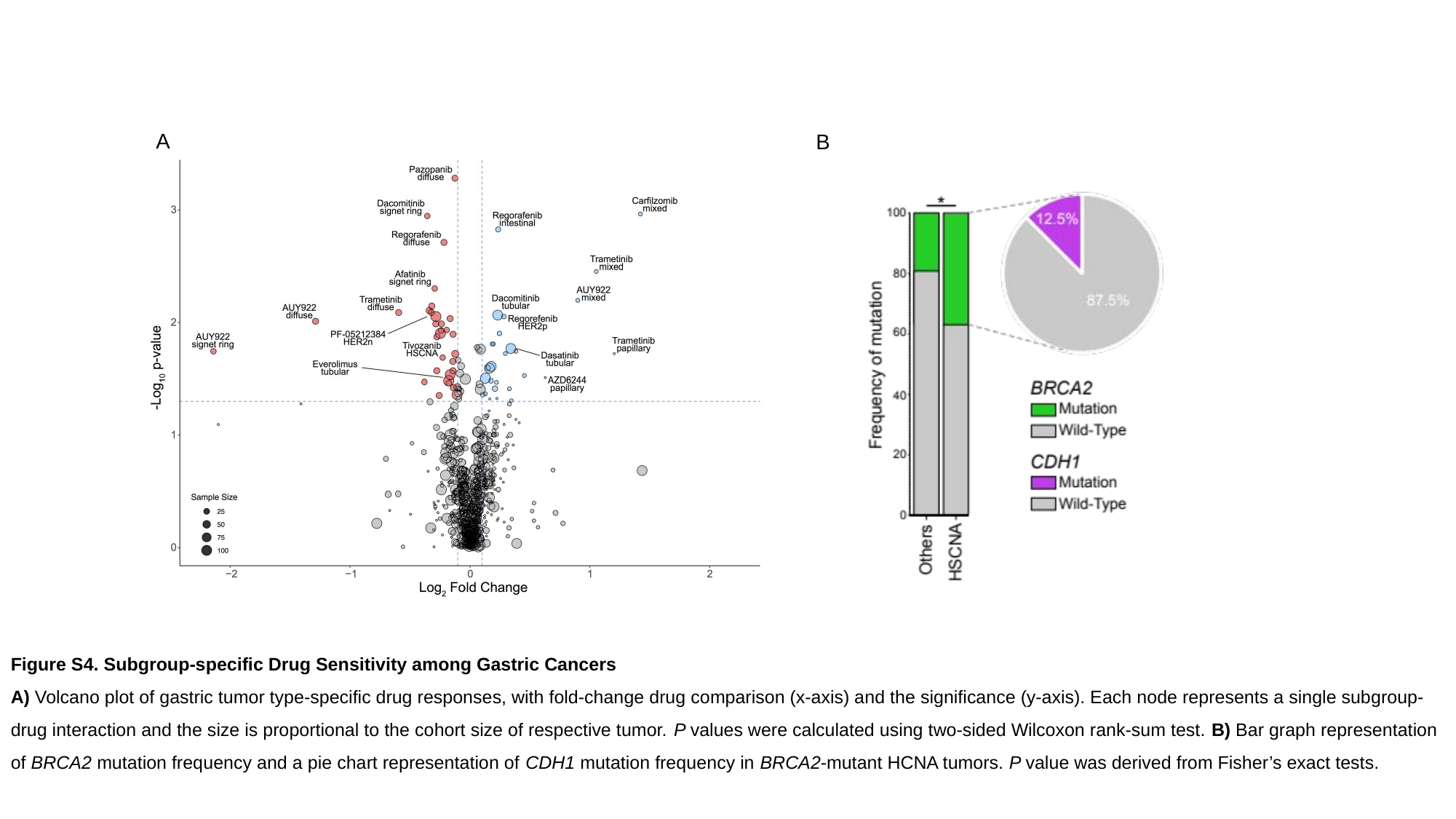

A
B
Figure S4. Subgroup-specific Drug Sensitivity among Gastric Cancers
A) Volcano plot of gastric tumor type-specific drug responses, with fold-change drug comparison (x-axis) and the significance (y-axis). Each node represents a single subgroup-drug interaction and the size is proportional to the cohort size of respective tumor. P values were calculated using two-sided Wilcoxon rank-sum test. B) Bar graph representation of BRCA2 mutation frequency and a pie chart representation of CDH1 mutation frequency in BRCA2-mutant HCNA tumors. P value was derived from Fisher’s exact tests.

## Slide 5
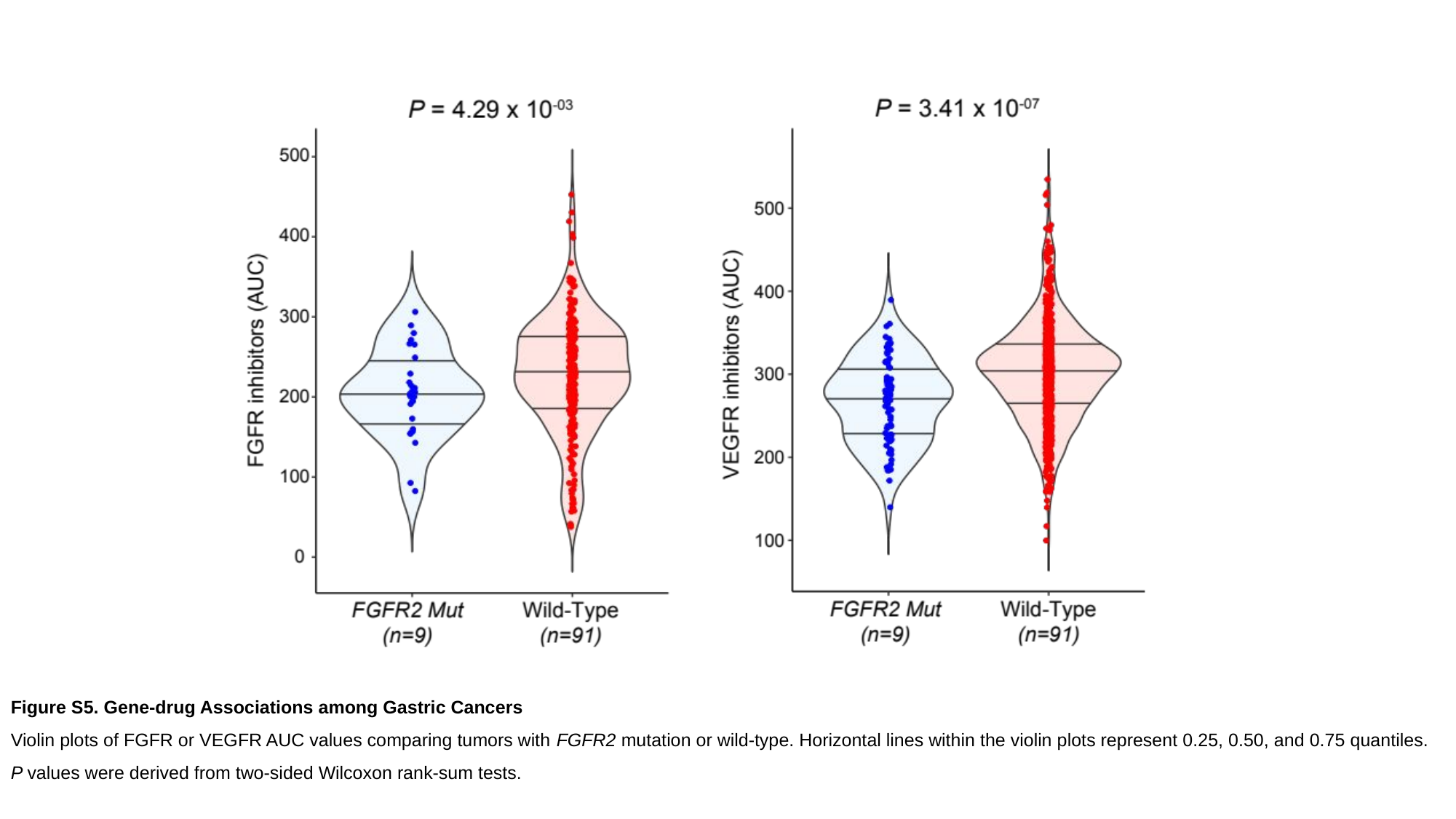

Figure S5. Gene-drug Associations among Gastric Cancers
Violin plots of FGFR or VEGFR AUC values comparing tumors with FGFR2 mutation or wild-type. Horizontal lines within the violin plots represent 0.25, 0.50, and 0.75 quantiles. P values were derived from two-sided Wilcoxon rank-sum tests.

## Slide 6
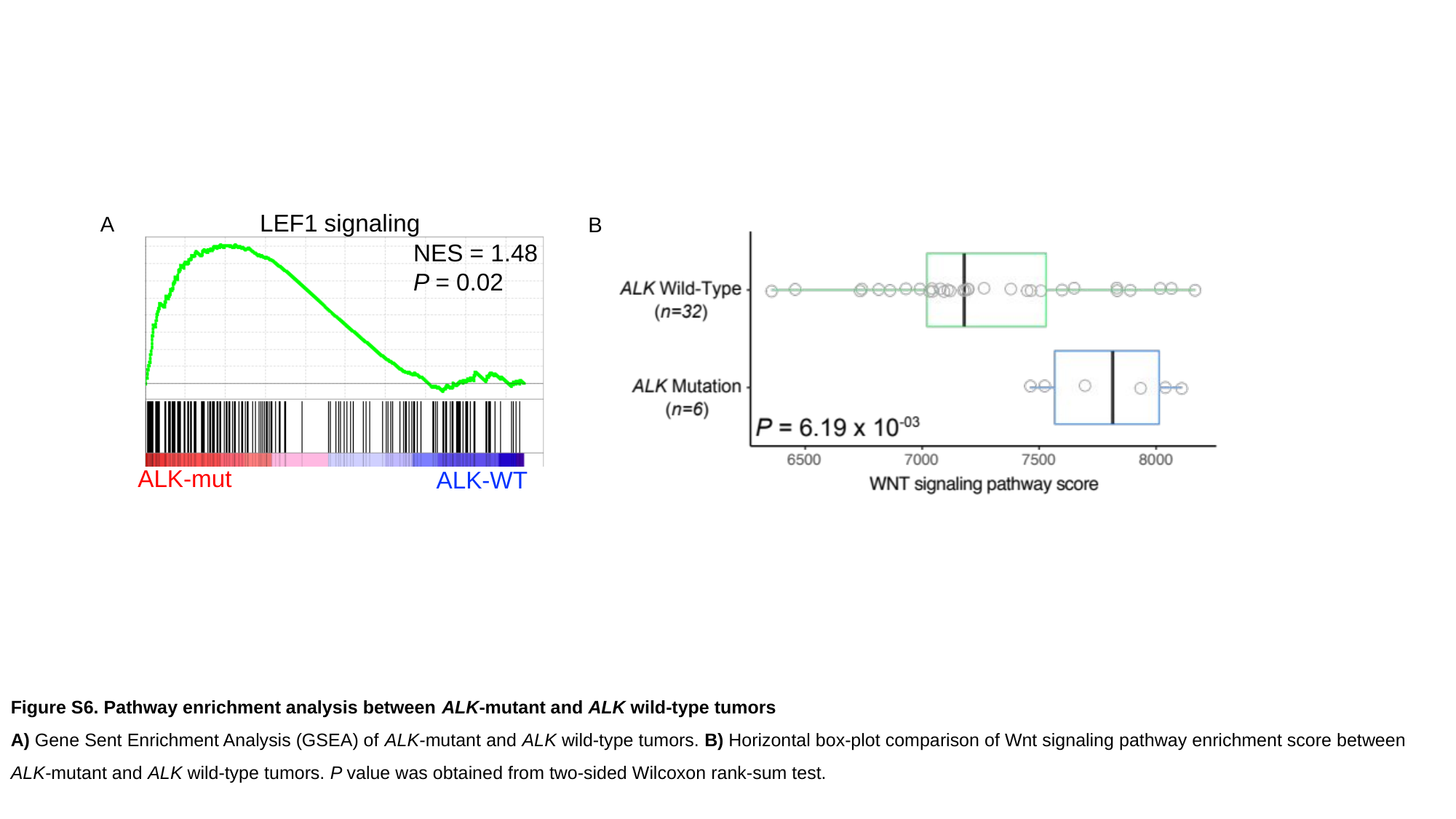

LEF1 signaling
NES = 1.48
P = 0.02
ALK-mut
ALK-WT
A
B
Figure S6. Pathway enrichment analysis between ALK-mutant and ALK wild-type tumors
A) Gene Sent Enrichment Analysis (GSEA) of ALK-mutant and ALK wild-type tumors. B) Horizontal box-plot comparison of Wnt signaling pathway enrichment score between ALK-mutant and ALK wild-type tumors. P value was obtained from two-sided Wilcoxon rank-sum test.

## Slide 7
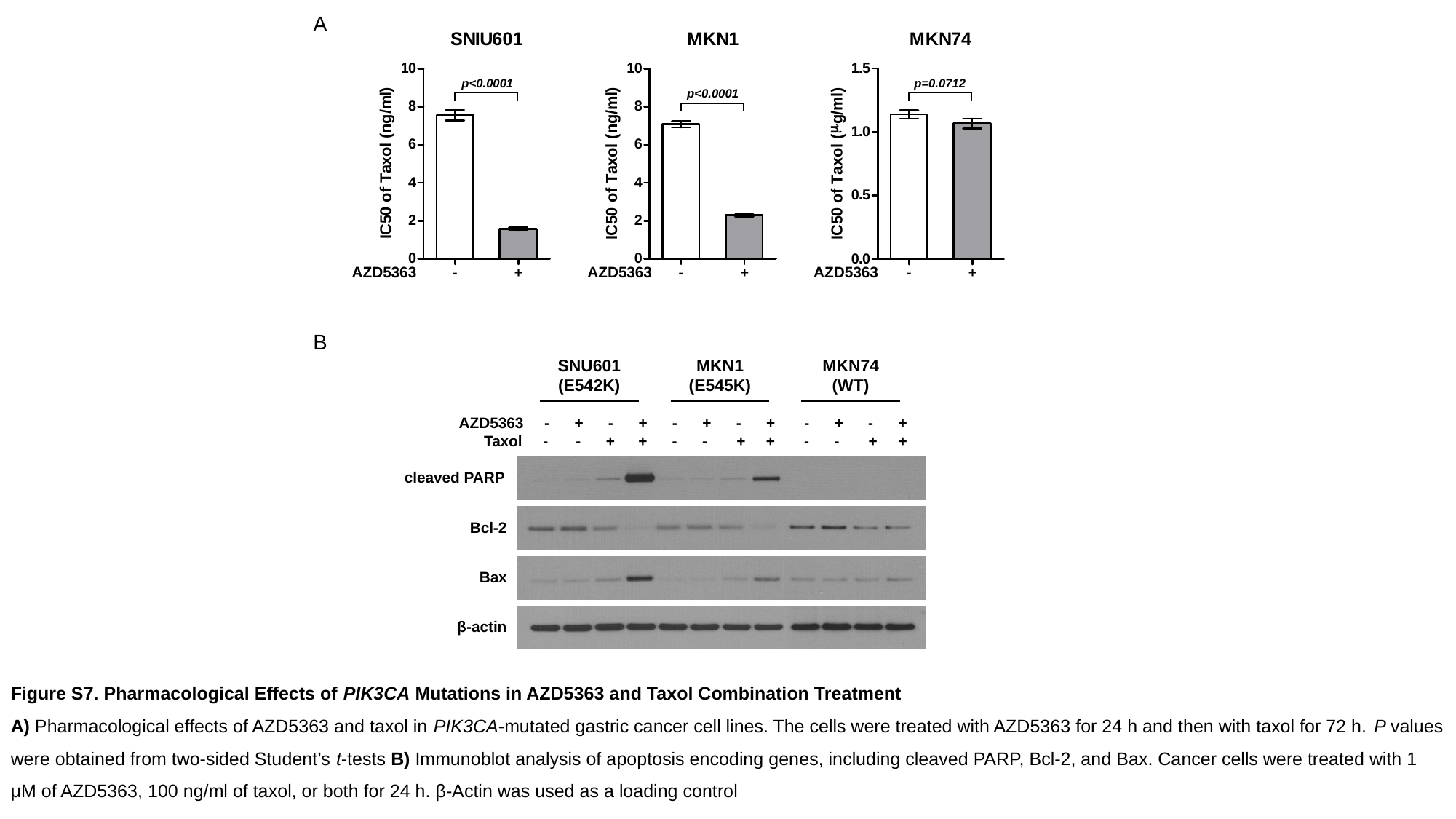

A
p<0.0001
AZD5363
p<0.0001
AZD5363
p=0.0712
AZD5363
B
SNU601
(E542K)
MKN1
(E545K)
MKN74
(WT)
AZD5363 - + - + - + - + - + - +
 Taxol - - + + - - + + - - + +
cleaved PARP
Bcl-2
Bax
β-actin
Figure S7. Pharmacological Effects of PIK3CA Mutations in AZD5363 and Taxol Combination Treatment
A) Pharmacological effects of AZD5363 and taxol in PIK3CA-mutated gastric cancer cell lines. The cells were treated with AZD5363 for 24 h and then with taxol for 72 h. P values were obtained from two-sided Student’s t-tests B) Immunoblot analysis of apoptosis encoding genes, including cleaved PARP, Bcl-2, and Bax. Cancer cells were treated with 1 μM of AZD5363, 100 ng/ml of taxol, or both for 24 h. β-Actin was used as a loading control

## Slide 8
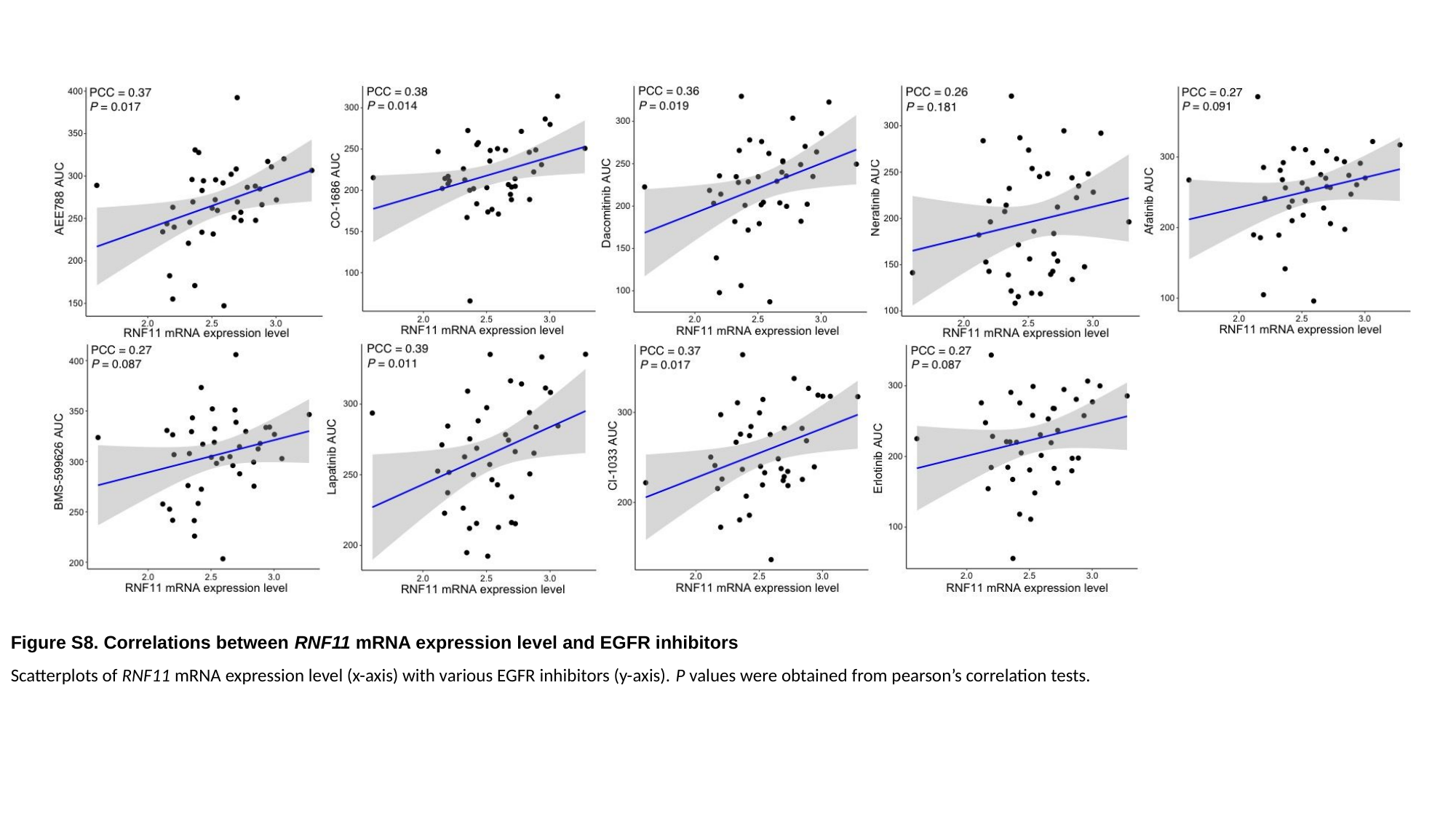

Figure S8. Correlations between RNF11 mRNA expression level and EGFR inhibitors
Scatterplots of RNF11 mRNA expression level (x-axis) with various EGFR inhibitors (y-axis). P values were obtained from pearson’s correlation tests.

## Slide 9
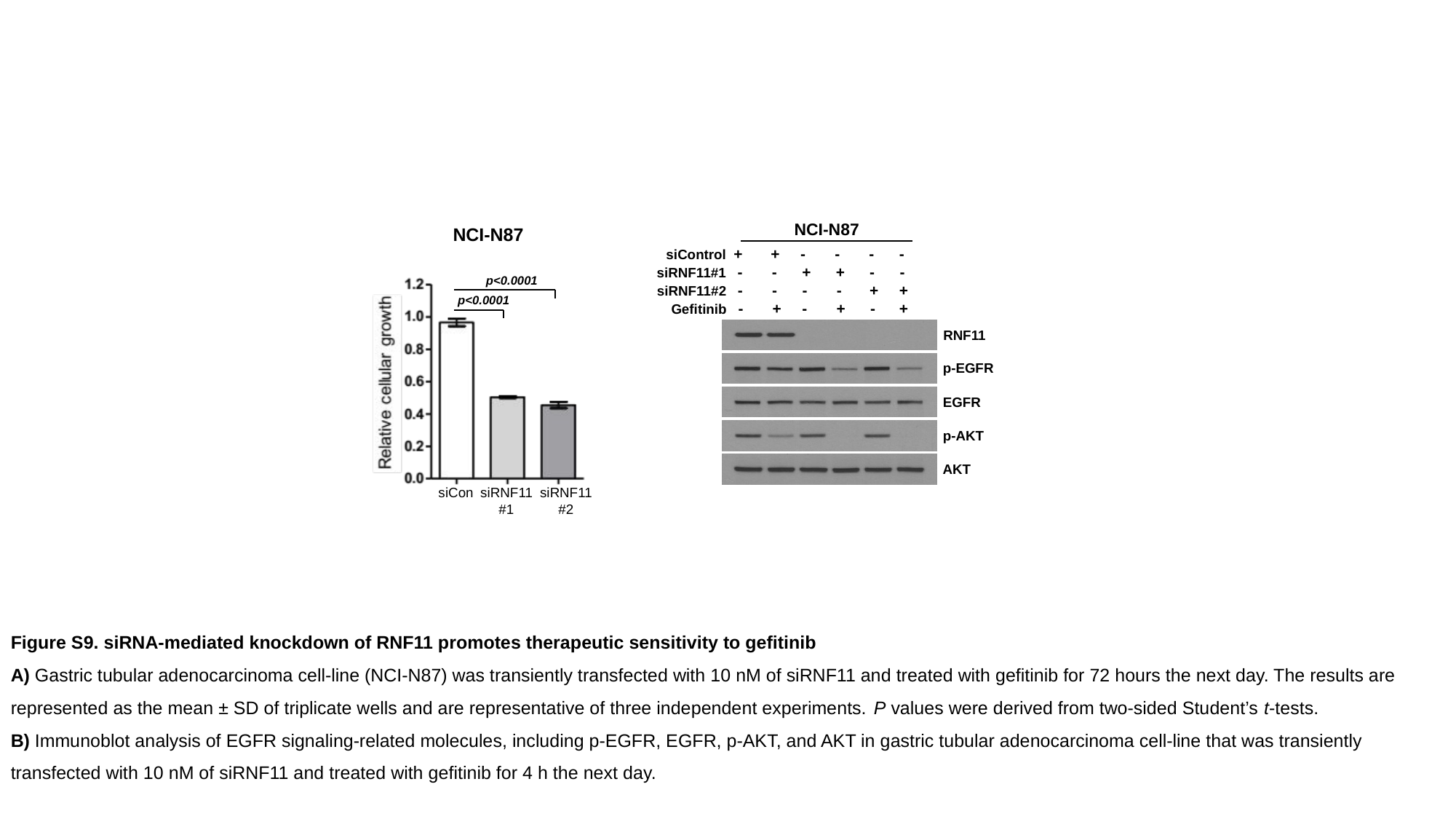

NCI-N87
siControl + + - - - -
siRNF11#1 - - + + - -
siRNF11#2 - - - - + +
Gefitinib - + - + - +
RNF11
p-EGFR
EGFR
p-AKT
AKT
NCI-N87
p<0.0001
p<0.0001
siCon
siRNF11
#1
siRNF11
#2
Figure S9. siRNA-mediated knockdown of RNF11 promotes therapeutic sensitivity to gefitinib
A) Gastric tubular adenocarcinoma cell-line (NCI-N87) was transiently transfected with 10 nM of siRNF11 and treated with gefitinib for 72 hours the next day. The results are represented as the mean ± SD of triplicate wells and are representative of three independent experiments. P values were derived from two-sided Student’s t-tests.
B) Immunoblot analysis of EGFR signaling-related molecules, including p-EGFR, EGFR, p-AKT, and AKT in gastric tubular adenocarcinoma cell-line that was transiently transfected with 10 nM of siRNF11 and treated with gefitinib for 4 h the next day.
